# Effects of Raspberry Leaf Tea Polyphenols on Postprandial Glucose and Insulin Responses in Healthy Adults

**DOI:** 10.3390/nu17172849

**Published:** 2025-09-01

**Authors:** Hind Mesfer S. Alkhudaydi, Jeremy P. E. Spencer

**Affiliations:** 1 Department of Food and Nutritional Sciences, School of Chemistry, Food and Pharmacy, University of Reading, Reading RG6 6DZ, UK; 2 Food Science and Nutrition Department, Faculty of Science, Taif University, Taif 26571, Saudi Arabia

**Keywords:** raspberry leaf tea, polyphenols, postprandial glucose, insulin response, sucrose metabolism, α-glucosidase inhibition, ellagic acid, dietary flavonoids, glycemic control, functional foods

## Abstract

**Background:** Dietary polyphenols, particularly flavonoids, have been associated with improved glycemic control and reduced risk of type 2 diabetes. Raspberry leaf (RL) is a rich but underexplored source of such bioactives, including ellagitannins, flavonoids, and phenolic acids. While raspberry fruit has received some attention in nutritional science, the metabolic effects of raspberry leaf—especially its influence on postprandial glucose and insulin responses—remain largely unstudied. **Objective:** This study is the first to investigate the acute effects of RL tea consumption on postprandial blood glucose and insulin levels in healthy individuals following intake of common dietary carbohydrates (sucrose and glucose). **Methods:** In a randomized crossover study, 22 healthy adults (12 males, 10 females) consumed 50 g of glucose or sucrose with or without 10 g of RL tea in four separate sessions. Blood glucose and insulin levels were measured at fasting and at 15, 30, 60, 90, and 120 min post-ingestion. A total of 37 polyphenolic compounds were identified in the RL infusion using LC–MS, following a 5-minute hot water extraction. The contents of ellagitannins, flavonoids, and phenolic acids were 38 mg, 7 mg, and 4 mg per 10 g of RL, respectively, contributing to a total polyphenol content of 50 mg per 10 g. **Results:** When RL tea was consumed with sucrose, postprandial blood glucose levels were significantly reduced at 15 and 30 min by 1.19 ± 0.88 mmol/L (25.59% reduction, *p* = 0.001) and 2.03 ± 1.05 mmol/L (43.57% reduction, *p* = 0.0004), respectively. Insulin concentrations were also significantly lower at 15 min (113.90 ± 59.58 pmol/L, *p* = 0.019), 30 min (161.76 ± 91.96 pmol/L, *p* = 0.0008), and 60 min (139.44 ± 75.96 pmol/L, *p* = 0.025). No significant differences were observed with glucose ingestion. **Conclusions:** This study provides the first clinical evidence that RL tea can blunt early postprandial glycemic and insulinemic responses to sucrose in healthy individuals. The data suggest that these effects are likely mediated by relatively low levels of polyphenols—particularly ellagic acid—through inhibition of carbohydrate-digesting enzymes such as α-glucosidase and β-fructofuranosidase. These findings support the potential of RL tea as a simple, dietary approach to modulate glucose metabolism and warrant further investigation in populations at risk for metabolic disorders.

## 1. Introduction

Type 2 diabetes mellitus (T2DM) is a major global health concern, with a dramatic rise in cases over the years [1,2]. According to the World Health Organization, diabetes—a long-term condition characterized by the body’s inability to produce sufficient insulin or use it efficiently, leading to high blood sugar levels—has seen its global prevalence double from 7% in 1990 to 14% in 2022, with over 800 million people affected [3]. If left untreated, comorbid conditions such as neuropathy, nephropathy, visual impairment, erectile dysfunction, heart disease, stroke, and peripheral vascular disease can lead to diabetic complications, including ulcers, gangrene, and amputation. Effective management of blood sugar levels is crucial in preventing such severe complications, including neuropathy, nephropathy, visual impairment, erectile dysfunction, cardiovascular disease, stroke, and peripheral vascular disease, which significantly increase the risk of developing severe diabetic complications, such as chronic ulcers, gangrene, and limb amputation [4]. Researchers have increasingly focused on developing alternative, low-risk therapeutic approaches instead of antidiabetic medications, based on the belief that effective blood glucose control can prevent and mitigate the clinical complications associated with T2DM [5].

Numerous naturally occurring plant-derived compounds have recently shown antidiabetic properties while also promoting overall health. For example, polyphenols are natural compounds recognized for their antioxidant and anti-inflammatory properties, contributing to the colors and flavors of fruits, vegetables, and teas [6]. These compounds, including flavonoids, tannins, and phenolic acids, offer various physiological benefits such as antidiabetic, antioxidant, anti-inflammatory, anticarcinogenic, and anti-obesity effects, making them instrumental in preventing chronic diseases like diabetes [7,8]. Consuming polyphenol-rich foods, such as tea, fruits, vegetables, chocolate, and red wine, regularly has been shown to alleviate oxidative stress and significantly reduce the risk of developing diabetes [9,10,11,12,13,14]. According to data from the UK Registry, a higher intake of anthocyanins and flavones was significantly associated with reduced insulin resistance and lower fasting insulin levels in females aged 18 to 76 [15]. Polyphenols enhance insulin sensitivity by alleviating oxidative stress in pancreatic cells, improving insulin secretion [16,17]. They further strengthen insulin signaling within cells by activating insulin receptor-associated proteins, such as AMP-activated kinase (AMPK), a key enzyme in glucose and energy regulation [18]. Studies have also demonstrated that polyphenols decrease glucose absorption in the intestine and slow digestion, thereby reducing post-meal blood sugar spikes [19,20,21,22].

Rubus idaeus (red raspberry), a member of the Rosaceae family with a long history of cultivation and global farming, includes around 700 species native to temperate regions and is widely used in traditional medicine [23,24,25]. RL is rich in nutrients and has long been used in traditional medicine to treat various ailments including diseases of the alimentary canal, airway and heart, and cardiovascular system, and can induce labor. RL has been recognized for its therapeutic properties, with Rubus idaeus green leaves listed in the British Pharmacopoeia since 1983 [26,27,28,29]. Raspberry leaves are a valuable source of bioactive compounds with notable antioxidants and antibacterial properties. They also contain appreciable levels of essential vitamins and minerals, including phosphorus, potassium, calcium, magnesium, and iron, along with ellagitannins, anthocyanins, and a diverse range of other polyphenolic compounds [30,31,32].

Currently, only 11 studies [33,34,35,36,37,38,39,40,41,42] have analyzed RL and identified a total of 52 compounds. Additionally, three studies reported that ellagic acid (EA) was the dominant compound in RL, with concentrations ranging from 292.20 to 438 mg/100 g [33,34,35]. The second most abundant compound was quercetin-3-O-galactoside, with concentrations ranging from 3.64 to 755 mg/100 g [36,37], followed by chlorogenic acid, which ranges from 22.94 to 104.16 mg/100 g [33,35,36,37,38]. RLs, rich in polyphenols such as ellagitannins, quercetin, and kaempferol derivatives, possess strong antioxidant, anti-inflammatory, and anti-diabetic properties, along with the ability to improve obesity and modulate gut microbiota, highlighting their considerable healthcare and industrial value [43,44,45,46,47]. Among the 25 types of ellagitannins identified in unripe raspberry (Rubus chingii), Chingiitannin A exhibited the most potent inhibitory activity against α-amylase and α-glucosidase [43]. The relationship between RL consumption and blood glucose levels is not well established due to limited scientific research. However, a notable case report from 2016 documented a 38-year-old woman with gestational diabetes mellitus who experienced hypoglycemia after consuming RL tea [48]. This suggests that RL may influence blood glucose levels, potentially reducing insulin requirements [48].

Given the lack of comprehensive studies—particularly randomized controlled trials—the effects of RL on blood glucose regulation remain largely unexplored. This study addresses a critical knowledge gap by investigating the potential health benefits of RL, which is rich in polyphenolic compounds such as flavonoids and ellagitannins known for their antioxidant properties and inhibitory effects on carbohydrate-digesting enzymes like sucrase. These bioactive compounds may modulate postprandial glucose absorption, offering a natural, plant-based approach to glycemic control. As the global burden of chronic diseases like type 2 diabetes continues to rise, there is an urgent need for affordable, effective, and sustainable interventions. Nutritional strategies involving polyphenol-rich functional foods are increasingly recognized as promising alternatives or complements to pharmaceutical treatments. RL tea, being widely accessible and inexpensive, has the potential to serve as a cost-effective dietary tool for improving glycemic responses. This study is the first to evaluate the acute effects of RL tea on postprandial blood glucose and insulin levels in healthy adults using a randomized, controlled, crossover design. We hypothesized that RL tea consumed alongside common dietary carbohydrates (sucrose and glucose) would attenuate postprandial increases in blood glucose and insulin. To test this, we measured glucose and insulin levels at multiple time points following ingestion of 50 g of either sucrose or glucose, with or without 10 g of RL tea infused in water.

## 2. Materials and Methods

### 2.1. Brewing Conditions and Polyphenol Analysis of Raspberry Leaf Tea

We and others have investigated the impact of steeping time on the extraction efficiency of polyphenols from RL tea using LC–MS [33]. In our previous study, tea infusions were prepared by steeping 2 g of dried RL leaves in 200 mL of boiling water for durations ranging from 0.5 to 20 min, replicating typical consumer practices. Each point was analyzed in triplicate to ensure reliability [33]. A total of 37 polyphenolic compounds were identified and quantified, with a focus on ellagitannins, flavonoids, and phenolic acids [33]. The aim of the study was to evaluate the concentrations of these compounds across different infusion times and to identify the optimal steeping duration for maximum polyphenol extraction [33].

### 2.2. In Human Study

#### 2.2.1. Study Participants

This randomized, crossover study, consisting of four visits, recruited 22 healthy volunteers from the Whiteknights Campus of the University of Reading. The study was conducted within the Hugh Sinclair Unit of Human Nutrition, part of the Department of Food and Nutritional Sciences at the University of Reading, between January 2024 and October 2024. The study aimed to assess the metabolic effects of dietary interventions in a controlled setting. Participants were included based on specific eligibility criteria. Volunteers aged between 18 and 65 years with a body mass index (BMI) ranging from 18 to 34.9 kg/m^2^ and fasting blood glucose levels between 3.9 and 5.5 mmol/L (70–100 mg/dL) were eligible to participate. They were required to be in good health, have no prior diagnosis of diabetes or prediabetes, and not be using medications for insulin resistance or diabetes. Additionally, participants had to provide written informed consent before enrolling in the study. Exclusion criteria were designed to minimize confounding factors and risks to participants. Individuals were excluded if they had abnormal results in renal function tests (e.g., blood urea nitrogen, creatinine), liver function tests (e.g., alanine transaminase, aspartate transaminase), or blood lipid profiles (e.g., cholesterol, triglycerides, high-density lipoprotein, low-density lipoprotein). Other exclusion factors included smoking, using certain medications (e.g., those for hypertension, hyperlipidemia, inflammation, or depression), and dietary supplements like cholesterol-lowering agents, fish oil, probiotics, prebiotics, or natural laxatives. Participants who had taken antibiotics within three months, consumed more than 14 units of alcohol per week, or had food allergies were also excluded. Further exclusions applied to individuals with chronic illnesses, severe impairments in heart, liver, or kidney function, or conditions such as celiac disease or other gastrointestinal, metabolic, neurological, or psychological disorders. Pregnant or breastfeeding women, those planning pregnancy within six months, and participants engaged in recent weight loss programs, herbal medication use, or other clinical trials were also deemed ineligible. Additionally, a history of malignancy, drug or alcohol dependency, or known allergies to components of the dietary intervention were disqualifying factors. The University of Reading Ethics Committee (UREC ID: 23_15, Approval Date: 18 December 2023) approved the study protocol, informed consent form, and sponsor qualifications. Written informed consent was obtained from all participants prior to screening. The study was registered in the Clinical Trials Registry under the identifier NCT06385626 on 26 April 2024. Participants who completed all study visits were compensated financially, with prorated compensation provided for those who did not complete the entire study.

#### 2.2.2. Sample Size Determination

Based on the findings of [16], the sample size was calculated to detect a reduction in glucose levels of 0.5 ± 0.6 mmol/L with a power of 0.8 (1-ꞵ) and a significance level (α) of 0.05. This calculation determined that 22 adults aged 18–65 would be required for the study. Considering an expected dropout rate of approximately 10% over the 2–4-month study duration, the goal was to retain at least 20 participants with complete primary outcome data. A final sample size of 20 participants per group was deemed adequate for statistically significant comparisons of diabetes-related outcomes, including blood glucose and insulin levels.

#### 2.2.3. Study Design

**Figure 1:**
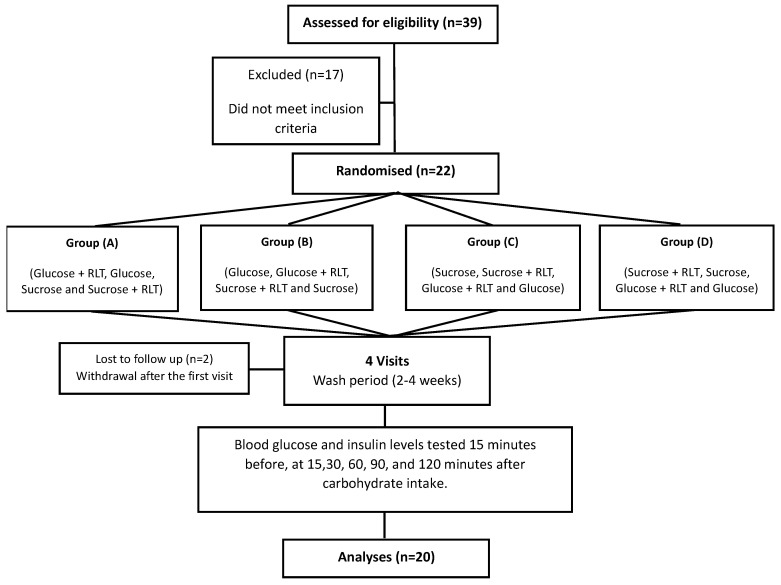
Patient disposition.

Using Stata 19 software and a randomized block design, a total of 22 participants were randomly assigned to one of four groups (A–D; see Figure 1). Five participants were distributed across groups, with two receiving six participants each. All participants were scheduled to undergo four interventions across four test sessions: glucose with or without RL tea and sucrose with or without RL tea. The order of these interventions was determined by the group to which the participants were assigned (Figure 1). Both participants and investigators were aware of the specific interventions administered during each test session; blinding was not implemented.

#### 2.2.4. Study Products

The study used Botanical World-branded RL tea collected in the United Kingdom. A polyphenol content analysis of the tea was conducted using LC–MS, comparing it to other teas from various European countries [33]. The tea from the UK showed superior polyphenol content (Table 1 summarizes the polyphenol content in 10 g of tea) [33]. The RL tea was prepared by grinding the leaves into a fine powder. To make the tea, 200 mL of Buxton water (Nestlé Waters UK Ltd., Haxby Road, York, North Yorkshire, YO31 8TA, UK) was boiled to 100 °C, and 10 g of tea was steeped for 5 min. The tea was then filtered completely using paper filters, after which sucrose or glucose was added as needed. Glucose and sucrose were used as control foods, while RL tea combinations were utilized as test foods. Sucrose (99.6% purity) and glucose (99.8% purity) were sourced from Ingredion. For participant consumption, 50 g of carbohydrate powder (glucose or sucrose) was dissolved with or without 10 g of RL tea (containing approximately 50 mg of polyphenols per 10 g). Each gram of glucose or sucrose provided 4 kcal (16.75 kJ) of energy.

#### 2.2.5. Procedures for Each Visit

Before each visit, participants were instructed to avoid excessive alcohol consumption, ensure adequate sleep, and maintain proper hydration. They were also required to fast from food and beverages, except water, for 8–10 h. Compliance with these requirements was confirmed before proceeding with the visit. Each participant arrived at the Hugh Sinclair Unit of Human Nutrition at 8:00 a.m. On arrival, they consumed the assigned test meal orally (carbohydrates with or without RL tea dissolved in 200 mL of warm water) within 5 min. Participants were then required to fast for 2 h, during which only water was allowed. Blood glucose and insulin concentrations were measured at baseline (15 min before the test meal) and 15, 30, 60, 90, and 120 min post-consumption. For each measurement, 5 mL of blood was collected via venipuncture. Each participant completed four test sessions on separate visits throughout the study (Figure 1). A 2–4-week washout period was implemented between sessions to eliminate any carryover effects from previous interventions and allow vein recovery. Glucose concentrations were measured using the Daytona Plus analyzer, and insulin levels were assessed using the Ella system, ensuring the accuracy and reliability of the collected data.

#### 2.2.6. Blood Sample Collection, Processing, and Biochemical Analysis

Blood samples were collected at each withdrawal using 5 mL yellow-top vacutainer tubes and transported in sealed containers to the Hugh Sinclair Unit of Human Nutrition (University of Reading). Within the laboratory, whole blood was centrifuged at 1750× *g* (~3000 rpm) for 15 min at room temperature. The separated serum, plasma, and buffy coat were carefully aspirated into labeled aliquot tubes and stored at –20 °C in designated storage boxes until all study visits were completed. Biochemical analyses were, therefore, performed in batch at the end of the collection period rather than on the day of collection.

Glucose concentrations were determined using the Randox RX Daytona Plus clinical chemistry analyzer with the hexokinase method (Randox, Cat. No. GL8319, Northern Ireland), following the manufacturer’s instructions. Samples were thawed, centrifuged briefly to remove air bubbles, and checked for clarity prior to analysis. The analyzer was routinely calibrated with multi-analyte calibrators, and quality control was monitored using commercial control sera at both normal and pathological levels. Internal QC results were plotted on Levey–Jennings charts with Westgard rules applied to detect bias or imprecision, and accuracy was externally validated through participation in the RIQAS scheme. Additional onboard safeguards, including reagent blanking, cuvette integrity checks, and liquid level sensing, were applied throughout the analysis.

Insulin concentrations were measured using the Ella automated immunoassay system (Protein Simple, UK) with Simple Plex Cartridge Kits. Serum samples were centrifuged at 8000–10,000× *g* for 4 min to remove particulates, diluted with assay buffer, and loaded into the cartridges together with wash buffer and assay controls, according to the manufacturer’s protocol. Calibration was performed using lot-specific factory calibration curves, and manufacturer-provided lyophilized high- and low-quality controls were reconstituted and included in each run to verify assay performance.

Together, these procedures—including strict pre-analytical handling, routine calibration, internal and external quality controls, and instrument safeguards—ensured the accuracy, reliability, and reproducibility of glucose and insulin measurements.

#### 2.2.7. Statistical Analysis

All statistical analyses were performed using SPSS software, version 28. Data are presented as mean ± standard deviation, with 95% confidence intervals (95% CI), or as median with a range where appropriate. Differences between multiple groups were analyzed using one-way analysis of variance (ANOVA). Post hoc tests were performed using Bonferroni correction if the homogeneity of variances was met or the Games–Howell test if variances were unequal. For comparisons between the two groups, the independent samples *t*-test was employed. Statistical significance was defined as *p* < 0.05.

## 3. Results

### 3.1. LC–MS/MS Analysis of Polyphenol Compounds in Raspberry Leaf Samples

In our previous study [33], we and others analyzed RL samples sourced from six distinct geographical regions to investigate the polyphenolic composition of aqueous extracts. Of the 52 targeted polyphenolic compounds, 37 were consistently detected across the samples [33]. Among the various steeping times assessed, a 5-minute infusion yielded the highest total polyphenol content (505.65 mg/100 g, *p* < 0.001), significantly exceeding the levels extracted at both shorter (409.84 mg/100 g) and longer durations (429.28 mg/100 g) [33]. Notably, EA reached its peak concentration at 5 min (380.29 mg/100 g), while phenolic acids were most abundant after 15 min (50.96 mg/100 g), and flavonoid content peaked at 4 min (82.58 mg/100 g) [33]. As detailed in Table 1, the concentrations of individual compounds are expressed in milligrams per 10 g of dried RL, serving as a standardized unit for reporting.

### 3.2. Basic Characteristics of Study Participants

Out of 39 healthy volunteers screened for inclusion, 17 individuals were excluded due to not meeting the inclusion criteria. Consequently, 22 participants (10 females and 12 males) were enrolled in the study. Of these, 20 participants completed all four visits along with the associated protocols. Two female participants withdrew after the first visit due to a lack of willingness to continue. The basic demographic and clinical characteristics of the participants who completed the study are presented in Table 2.

### 3.3. Blood Glucose and Insulin Levels Before and During Sucrose Intake with or Without RL Tea

**Figure 2:**
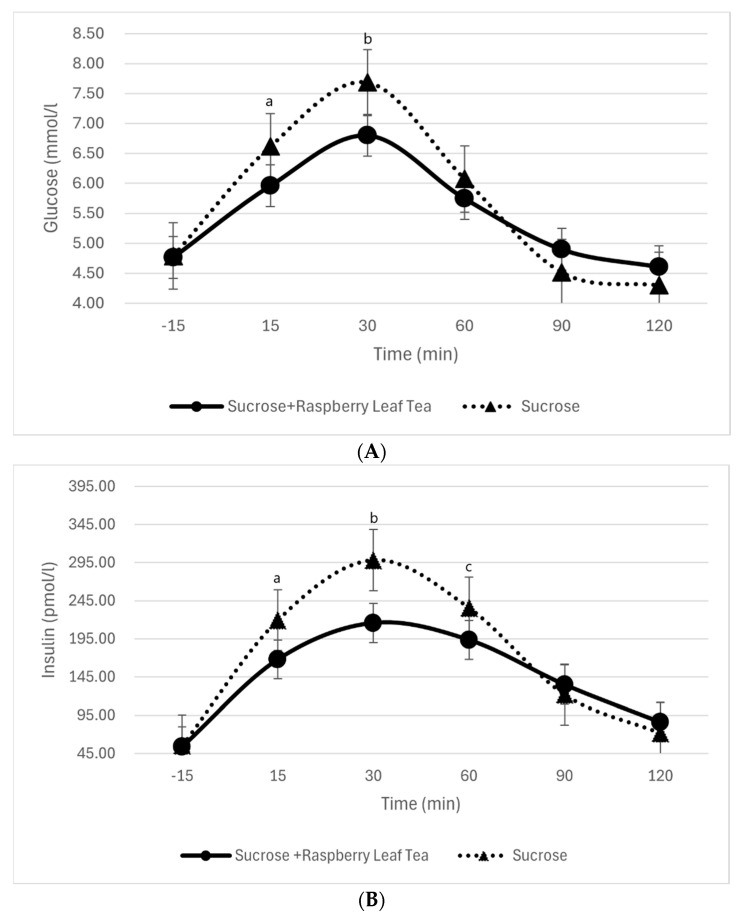
(**A**) Effect of raspberry leaf tea on sucrose-induced blood glucose response. Means that do not share the same letter are significantly different. For example, the sucrose + RLT peak was significantly lower than sucrose at 30 min (*p* = 0.001, label “a”) and at 60 min (*p* = 0.00008, label “b”). (**B**) Effect of raspberry leaf tea on sucrose-induced insulin response. Means with the same letter are not significantly different. Peaks marked with different letters indicate significant differences. For example, sucrose + RLT was significantly lower than sucrose at 15 min (*p* = 0.019, label “a”), at 30 min (*p* = 0.00008, label “b”), and at 60 min (*p* = 0.025, label “c”).

Baseline blood glucose levels, measured 15 min prior to the test meals, were comparable across all conditions (sucrose and glucose, with or without tea). Following the test meals, blood glucose levels increased gradually, peaking at 30 min post-intake, before declining back to baseline by 120 min. For sucrose, postprandial blood glucose levels at 15 and 30 min were significantly lower when consumed with tea compared to sucrose alone (*p* < 0.01), indicating a moderating effect of RL tea on the glycemic response. In contrast, for glucose, there was no statistically significant difference in blood glucose levels between consumption with or without tea, suggesting that the tea’s effect may vary based on the carbohydrate source. Insulin levels were significantly lower at 15, 30, and 60 min when consumed with RL tea compared to sucrose alone (*p* = 0.019, *p* = 0.0008, and *p* = 0.025, respectively). These findings demonstrate that RL tea effectively attenuates the insulin response to sucrose consumption, as shown in Table 3 and Table 4 and Figure 2A,B.

### 3.4. Blood Glucose and Insulin Levels Before and During Glucose Intake with or Without RL Tea

**Figure 3:**
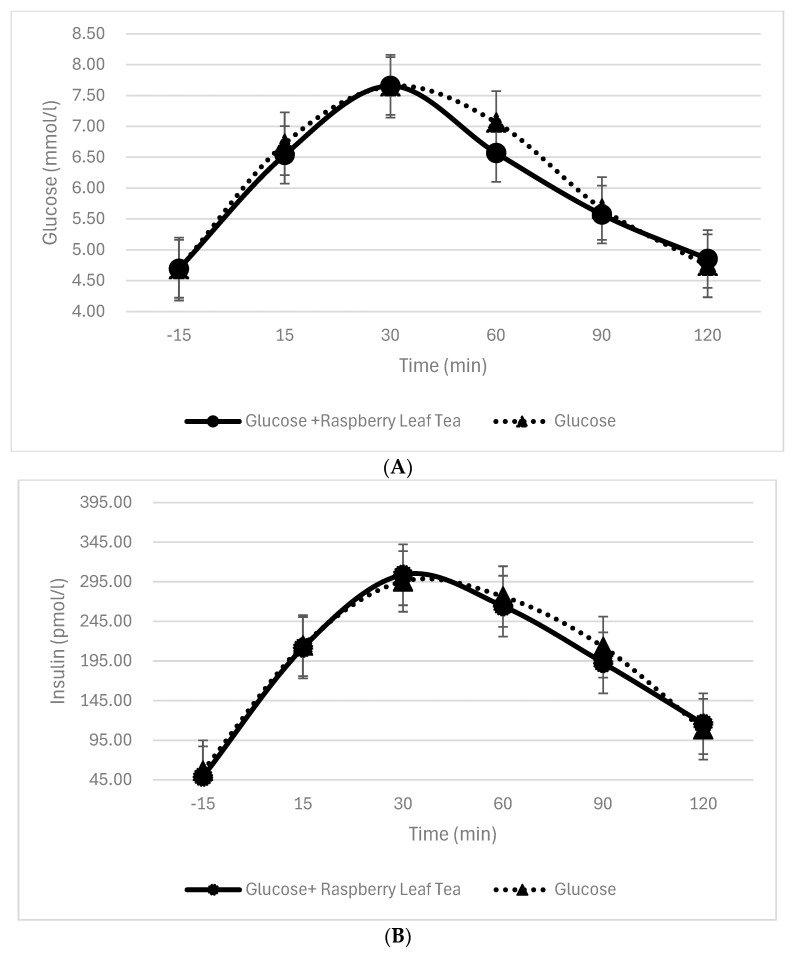
(**A**) Effect of raspberry leaf tea on glucose-induced blood glucose response. (**B**) Effect of raspberry leaf tea on glucose-induced insulin response.

Similar to the trend observed with blood glucose levels, insulin levels increased following the test meals, peaking at 30 min before gradually returning to baseline levels by 120 min. This pattern was consistent across all conditions, regardless of whether sucrose or glucose was consumed with or without RL tea. In contrast, for glucose, no statistically significant difference in insulin levels was observed at any time point between consumption with or without RL tea, as illustrated in Table 3 and Table 4 and Figure 3A,B. This indicates that the tea’s moderating effect on insulin response is specific to sucrose and does not extend to glucose.

## 4. Discussion

This study is the first to investigate the effects of red raspberry leaf (*Rubus idaeus*) consumption on postprandial blood glucose and insulin responses in healthy individuals. The findings indicate that components contained within the tea, released on brewing, reduce postprandial blood glucose and insulin levels following sucrose but not glucose ingestion. Considering the well-documented importance of controlling high peak glucose levels in the management of diabetes, these preliminary findings indicate the potential utility of RL to modulate postprandial blood glucose and insulin levels in response to carbohydrate intake, in this case sucrose. Mechanistically, this effect may be attributed, at least in part, to the inhibition of α-glucosidase enzymes, which are essential for the hydrolysis of complex carbohydrates into absorbable monosaccharides such as glucose—something observed for other polyphenol-rich plant extracts [49,50,51,52,53]. By delaying carbohydrate breakdown, glucosidase inhibitors reduce the rate of glucose absorption, thus lowering the glycemic index of meals. Importantly, the ingestion of RL in our study did not induce gastrointestinal side effects, such as diarrhea, which are commonly associated with pharmacological glucosidase inhibitors like acarbose [49]. Since sucrose—a disaccharide composed of glucose and fructose linked by an α-1,2-glycosidic bond—is hydrolyzed by both α-glucosidase and β-fructofuranosidase, the observed attenuation of glycamic response suggests a possible interaction between RL components and these enzymatic pathways [49].

The use of *Rubus idaeus* waste material has been considered for applications in the pharmaceutical and functional food industries due to its richness in bioactive compounds [54,55]. The RL tea used in this study delivered approximately 50 mg of total polyphenols per 10 g dose, including 38 mg ellagic acid, 6.4 mg quercetin-3-O-glucuronide, and 2 mg/g chlorogenic acid. While many studies on these compounds involve chronic supplementation, several acute trials provide relevant comparisons. Chlorogenic acid, in a randomized crossover trial involving overweight men, in a single dose of 1 g, significantly reduced glucose (−0.7 mmol/L, *p* = 0.007) and insulin (−73 pmol/L, *p* = 0.038) concentrations at 15 min post-OGTT [56]. However, no significant effects were observed on the overall glucose or insulin area under the curve compared to placebo [56]. Acutely administering chlorogenic acid at 100–1000 mg has also been shown to reduce postprandial glucose and insulin responses in healthy and obese individuals [57,58]. Although the chlorogenic acid content in our tea was considerably lower, its effect may be enhanced by the co-presence of other polyphenols. For example, in human intervention studies, quercetin has demonstrated mixed glycemic effects, and acute single-dose trials (e.g., onion-skin extract) did not show meaningful reduction in postprandial glucose or insulin [59]. Direct acute data on EA are limited, but berries rich in ellagitannins [60] (their precursors) offer indirect support. For instance, [61] demonstrated that consuming 150 g of mixed berry purée with 35 g sucrose significantly reduced early postprandial glucose and insulin concentrations in healthy subjects. A similar effect was observed when blackcurrant or lingonberry purée was consumed with sucrose, showing attenuated glucose and insulin peaks and improved glycemic stability over two hours [61]. These findings suggest that there may be more complex interactions at play. Notably, previous studies involved higher overall polyphenol intake than used in our study, suggesting that effects on carbohydrate digestion are possible at much lower intake levels, perhaps due to additive or synergistic effects on carbohydrate digestion and glucose absorption, or due to the effectiveness of ellagic acid. In addition, this may be because other compounds in RL tea have not yet been discovered that may influence blood glucose regulation. We previously analyzed 52 compounds [33], but further investigation is necessary to identify additional bioactive compounds.

The postprandial glucose-lowering effects of RL tea are likely mediated through inhibition of key digestive enzymes—particularly sucrase, maltase, and α-glucosidase—which hydrolyze dietary disaccharides into absorbable monosaccharides such as glucose and fructose [62]. Ellagic acid, the dominant polyphenol in our extract (38 mg/10 g), has shown strong α-glucosidase inhibitory activity, with reported IC_50_ values between 140.2, 191.4, and 380.9 μmol/L, depending on assay conditions [63]. It binds competitively to the enzyme’s active site through hydrogen bonding and hydrophobic interactions, inducing conformational changes and destabilizing enzymatic structure [64,65,66,67,68,69,70]. Quercetin-3-O-glucuronide (Q3GA), present at 6.4 mg per 10 g, is believed to exert reversible mixed-mode inhibition of α-glucosidase, with an IC_50_ of 108.11 ± 4.61 μM [67]. Though its effects on sucrase are less studied, flavanol conjugates like Q3GA are known to inhibit intestinal disaccharidases in vitro [71]. Chlorogenic acid, found at 2 mg/10 g in our extract, inhibits both intestinal α-glucosidase and hepatic glucose-6-phosphatase by binding key catalytic residues, thereby reducing both intestinal glucose release and hepatic gluconeogenesis [72,73]. These mechanisms collectively delay carbohydrate breakdown and glucose absorption, resulting in a flatter postprandial glucose curve and reduced early-phase insulin secretion [63]. Attenuating these peaks alleviates pressure on pancreatic β-cells, limits hepatic lipogenesis, and helps maintain peripheral insulin sensitivity [63]. Such glycemic modulation mirrors the action of pharmaceutical α-glucosidase inhibitors (e.g., acarbose) but occurs via naturally occurring dietary polyphenols such as ellagic acid, quercetin-3-O-glucuronide, and chlorogenic acid — supporting their potential use in maintaining metabolic health in both healthy and at-risk populations [64,65,66,67,68,69,70,71].

Polyphenols are widely recognized for their potential role in modulating glucose metabolism, with numerous clinical studies evaluating the effects of polyphenol-rich foods such as blueberries, cocoa, and green tea on glycemic control, insulin sensitivity, and cardiometabolic health [74]. While several trials have demonstrated improvements in these parameters [75,76,77,78,79,80,81,82,83,84,85,86,87,88,89,90,91,92,93,94], others have reported no significant metabolic changes [95,96,97,98,99,100], highlighting the variability of outcomes depending on compound type, dosage, and study design. In our acute study on healthy individuals, the observed reduction in postprandial glucose and insulin levels following RL intake may suggest that its polyphenolic content plays a role in short-term glycemic modulation. Among the polyphenols present in RL, EA is one of the most abundant and biologically active. Several randomized controlled trials have investigated its metabolic effects, with EA supplementation at 180 mg/day for 8 weeks resulting in significant improvements in fasting glucose, postprandial glucose, HbA1c, insulin resistance, and inflammatory and oxidative stress markers in individuals with type 2 diabetes [101,102,103,104]. Higher doses, administered at 500 mg/day over a 12-week period, have also produced favorable outcomes in individuals with metabolic syndrome, including improved insulin sensitivity and reductions in triglycerides, BMI, and blood pressure [105]. Although our study did not target diabetic populations or chronic outcomes, the findings align with the growing body of evidence supporting the acute metabolic effects of polyphenols—particularly ellagic acid—and suggest that RL may hold potential for glycemic regulation, warranting further investigation in both healthy and at-risk populations.

The strength of our study is reinforced by the use of venous blood sampling rather than fingerstick collection, as recent evidence [106,107] demonstrates that venous samples provide more accurate and reliable glucose measurements, likely due to reduced variability in perfusion and minimized influence from external factors such as temperature, pressure, and local tissue metabolism, which can affect capillary samples. This study has several limitations. First, its small sample size, short duration, and recruitment of only healthy adults aged 22–62 from a single UK location limit the generalizability of the findings across age groups, populations, and clinical conditions. Second, the study design focused on only two carbohydrate types (sucrose and glucose) and did not evaluate the glycemic impact of more complex carbohydrates or whole foods, nor did it assess long-term markers such as HbA1c or insulin resistance (e.g., HOMA-IR). Third, lifestyle factors—such as diet, physical activity, and habitual polyphenol intake—were not controlled for, and no food diaries were used, which may have introduced variability in participants’ nutritional status. Finally, the absence of a placebo group increases the risk of bias. Despite these limitations, we believe that our data show novel evidence for the impact of polyphenols, in this case present in RL tea on glucose absorption, at significantly lower concentrations than that observed previously.

Maintaining optimal postprandial blood glucose and insulin levels is critical for preventing type II diabetes. Our results suggest that RL may help blunt glycemic and insulinemic responses to disaccharides. This effect could be due to polyphenolic compounds in the leaf that inhibit carbohydrate-digesting enzymes or glucose transporters, thereby reducing sugar absorption in the gut. These findings support the potential of RL as a dietary strategy to modulate glucose metabolism. Future studies should explore the effects of RL in larger, more diverse populations, including individuals with diabetes or impaired glucose tolerance, and assess its impact over longer durations using clinical endpoints such as HbA1c and insulin resistance (HOMA-IR). Trials involving real-world dietary patterns, a wider range of carbohydrates, and placebo controls will also be essential to fully understand the clinical relevance and mechanisms of action of RL polyphenols.

## Figures and Tables

**Table 1:** Polyphenols content in RL (mg/10 g DW) [33].

Compound Identity	Content, mg/10 g
Epigallocatechin	0.02 ± 0.00
Epicatechin gallate	0.18 ± 0.14
Cyanidin Chloride	0.34 ± 0.05
Catechin	0.06 ± 0.01
Vanillin	0.06 ± 0.00
Epicatechin	0.36 ± 0.00
Naringenin	0.02 ± 0.00
Hesperetin	0.02 ± 0.00
Verbascoside	0.02 ± 0.00
Chlorogenic Acid	2.21 ± 0.09
Neochlorogenic Acid	0.05 ± 0.00
Cryptochloroqenic Acid	0.09 ± 0.01
Quercetin	0.00 ± 0.00
Myricetin	0.01 ± 0.00
P-Coumaric Aicd	0.09 ± 0.00
Salicylic Acid	0.03 ± 0.01
Vanillic Acid	0.15 ± 0.02
Mcoumaric	0.07 ± 0.00
Ocoumaric	0.01 ± 0.00
Isoferulic Acid	0.03 ± 0.00
Ellagic Acid	38.03 ± 3.59
Ferulic Acid	0.04 ± 0.00
Phloridizin	0.03 ± 0.05
Gallic Acid	0.68 ± 0.02
3.4-dihydroxybenzoic acid	0.18 ± 0.00
2,3-Dihydroxybenzoic Acid	0.25 ± 0.01
Caffeic Acid	0.35 ± 0.01
Luteolin-7-O-glucoside	0.09 ± 0.01
Quercetin-3-O-glucuronide	6.47 ± 0.43
Quercetin-3-O-rutinoside	0.24 ± 0.01
Quercetin 3-O-galactoside	0.14 ± 0.00
Kaempferol-3-O-glucoside	0.08 ± 0.01
Quercetin-3-glucoside	0.14 ± 0.00
Kaempferol-O-rutinoside	0.02 ± 0.00
Petunidin Chloride	0.01 ± 0.00
Gallocatechin	0.00 ± 0.00
Total of Ellagitannin content (mg/10 g)	38.03 ± 3.59
Total of Flavonoid content (mg/10 g)	7.76 ± 0.49
Total of Phenolic Acid content (mg/10 g)	4.78 ± 0.15
Total of Polyphenol content (mg/10 g)	50.56 ± 3.00

**Table 2:** Baseline characteristics of the study population.

Characteristic	N	Mean ± SD	95% CI	Range	Median
Age, year	20	34.86 ± 45.94	34.86–45.94	62–22	39.5
Height, cm	20	164.98 ± 174.92	164.98–174.92	192–153	170.5
Weight, kg	20	64.17 ± 76.9	64.17–76.9	104.5–53	63.7
BMI, kg/m^2^	20	22.82 ± 25.77	22.82–25.77	30.1–20	23.3
Fat %	20	20.81 ± 26.95	20.81–26.95	40–9.9	23.5
Fasting Blood Glucose (mmol/L)	20	4.43 ± 4.89	4.43–4.89	5.68–2.95	4.75
Fasting Blood Insulin (Pmol/L)	20	41.91 ± 59.74	41.91–59.74	91.8–21.6	44.7
HOMA-IR	20	1.43 ± 2.07	1.43–2.07	2.93–0.71	1.52
Systolic blood pressure, mmHg	20	115.1 ± 125.6	115.1–125.6	147–100	119
Diastolic blood pressure, mmHg	20	72.38 ± 79.82	72.38–79.82	96–65	73

BMI, body mass index; HOMA-IR, Homeostatic Model Assessment of Insulin Resistance; SD, standard deviation; 95% CI, 95% confidence interval.

**Table 3:** Postprandial blood glucose changes relative to preprandial levels across test and reference foods.

Food Group	Change in Postprandial Blood Glucose Versus 15 min Before Carbohydrates Intake (mmol/L and %)										
	n	15 min	%	30 min	%	60 min	%	90 min	%	120 min	%
Glucose	20	2.03 ± 0.95	43.30	2.98 ± 1.46	63.57	2.40 ± 1.72	51.13	1.02 ± 1.53	21.74	−0.031.05	−0.57
Glucose + RLT	20	1.93 ± 0.72	41.47	3.00 ± 1.01	64.59	2.06 ± 1.97	44.24	0.95 ± 1.80	20.43	0.03 ± 1.64	0.64
*p*		0.64		0.94		0.36		0.78		0.79	
Sucrose	20	1.83 ± 0.87	38.32	2.90 ± 0.95	60.69	1.29 ± 1.38	26.95	−0.27 ± 0.84	−5.70	−0.96 ± 2.18	−20.10
Sucrose + RLT	20	1.19 ± 0.88	25.59	2.03 ± 1.05	43.57	1.23 ± 1.29	26.36	0.13 ± 0.76	2.85	−0.16 ± 0.77	−3.44
*p*		0.001		0.0004		0.23		0.06		0.16	

RLT, Raspberry Leaf Tea; N, number. Data are presented as mean ± standard deviation (mean ± S.D.).

**Table 4:** Postprandial blood insulin changes relative to preprandial levels for test and reference foods.

Food Group	Change in Postprandial Blood Insulin Versus 15 min Before Carbohydrates Intake (pmol/L and %)										
	n	15 min	%	30 min	%	60 min	%	90 min	%	120 min	%
Glucose	20	159.90 ± 139.24	287.71	239.88 ± 95.30	431.62	214.48 ± 114.60	385.92	157.15 ± 146.51	282.77	53.47 ± 76.64	96.21
Glucose + RLT	20	162.79 ± 78.23	331.75	254.78 ± 103.43	519.22	215.10 ± 144.40	438.34	143.63 ± 130.22	292.69	61.34 ± 94.16	125.01
*p*		0.93		0.50		0.98		0.45		0.44	
Sucrose	20	164.07 ± 125.70	296.71	242.90 ± 125.01	439.28	180.25 ± 94.02	325.98	66.62 ± 58.57	120.48	16.40 ± 32.78	29.66
Sucrose + RLT	20	113.90 ± 59.58	211.05	161.76 ± 91.96	299.74	139.44 ± 75.96	258.39	80.84 ±58.40	149.79	31.87 ± 43.97	59.06
*p*		0.01		0.0008		0.02		0.21		0.12	

RLT, Raspberry Leaf Tea; N, number. Data are presented as mean ± standard deviation (mean ± S.D.).

## Data Availability

The data supporting the findings of this study are included within the article. Further inquiries can be directed to the corresponding author.
